# The Intestinal Eukaryotic Virome in Healthy and Diarrhoeic Neonatal Piglets

**DOI:** 10.1371/journal.pone.0151481

**Published:** 2016-03-16

**Authors:** Oskar E. Karlsson, Jenny Larsson, Juliette Hayer, Mikael Berg, Magdalena Jacobson

**Affiliations:** 1 Department of Biomedical Sciences and Veterinary Public Health (BVF), Swedish University of Agricultural Sciences (SLU), Uppsala, Sweden; 2 SLU Global Bioinformatics Centre, Department of Animal Breeding and Genetics (HGEN), SLU, Uppsala, Sweden; 3 The OIE Collaborating Centre for the Biotechnology-based Diagnosis of Infectious Diseases in Veterinary Medicine, Uppsala, Sweden; 4 Department of Clinical Sciences (KV), Swedish University of Agricultural Sciences (SLU), Uppsala, Sweden; Universiteit Utrecht, NETHERLANDS

## Abstract

Neonatal porcine diarrhoea of uncertain aetiology has been reported from a number of European countries. The aim of the present study was to use viral metagenomics to examine a potential viral involvement in this diarrhoea and to describe the intestinal virome with focus on eukaryotic viruses. Samples from the distal jejunum of 50 diarrhoeic and 19 healthy piglets from 10 affected herds were analysed. The viral fraction of the samples was isolated and nucleic acids (RNA and DNA fractions) were subjected to sequence independent amplification. Samples from diarrhoeic piglets from the same herds were pooled whereas samples from healthy piglets were analysed individually. In total, 29 clinical samples, plus two negative controls and one positive control consisting of a mock metagenome were sequenced using the Ion Torrent platform. The resulting sequence data was subjected to taxonomic classification using Kraken, Diamond and HMMER. In the healthy specimens, eight different mammalian virus families were detected (*Adenoviridae*, *Anelloviridae*, *Astroviridae*, *Caliciviridae*, *Circoviridae*, *Parvoviridae*, *Picornaviridae*, and *Reoviridae*) compared to four in the pooled diarrhoeic samples (*Anelloviridae*, *Circoviridae*, *Picornaviridae*, and *Reoviridae*). It was not possible to associate a particular virus family with the investigated diarrhoea. In conclusion, this study does not support the hypothesis that the investigated diarrhoea was caused by known mammalian viruses. The results do, however, indicate that known mammalian viruses were present in the intestine as early as 24–48 hours after birth, indicating immediate infection post-partum or possibly transplacental infection.

## Introduction

Neonatal porcine diarrhoea (NPD) is a common problem in pig farming worldwide and thus contributes to morbidity and mortality among piglets, particularly in intensive farming systems [1,2]. Hence, NPD has substantial adverse effects on both economical and animal welfare aspects of the pig industry [3]. The condition is associated with a number of well-known pathogens including bacterial agents, protozoa, and viruses such as rotavirus, transmissible gastroenteritis virus (TGEV), and porcine epidemic diarrhoea virus (PEDV) [2]. Additional viruses including astrovirus [4,5], bocaparvovirus [6], kobuvirus [7,8], sapovirus [9], and sapelovirus [10] have occasionally been associated with diarrhoea in piglets, although their clinical significance is yet to be established.

Reports from a number of countries, including Sweden, describe problems with NPD despite adequate vaccination- and management routines [11–13]. This condition, referred to as new neonatal porcine diarrhoea syndrome (NNPDS), does not seem to be associated with well-known pathogens and it is hypothesized that unexpected or divergent viruses, evading routine diagnostic testing, may be a contributing factor [14]. To investigate a potential viral involvement in this disease syndrome, an unbiased exploratory approach such as that offered by viral metagenomics, would be desirable [15,16]. Metagenomics is the study of metagenomes, defined as the genomes of all organisms present in a sample and offers the possibility of studying the whole microbial composition.

**Fig 1:**
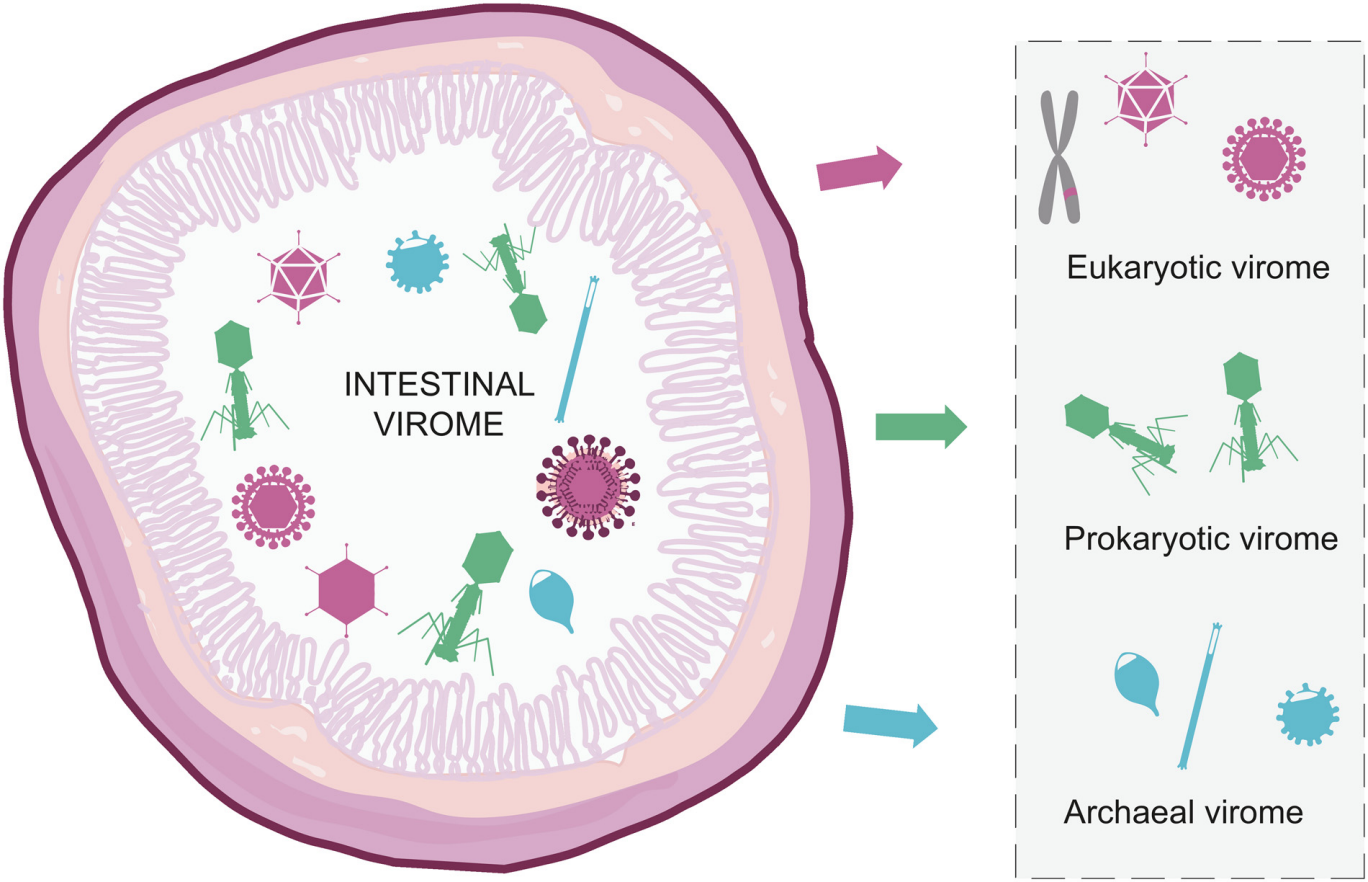
Schematic illustration of the intestinal virome. The mammalian intestinal virome is proposed to have three parts—the eukaryotic, the prokaryotic, and the archaeal. The eukaryotic virome includes host-infecting viruses, viruses infecting other eukaryotes in the intestine, and feed-associated viruses.

With the advent of metagenomics studies, the knowledge of the different components and the complexity of the microbiome greatly expanded [17,18]. The intestine represents a complex environment with both eukaryotic, prokaryotic, archaeal, and viral elements [19]. Hitherto, most studies on the intestinal microbiome have addressed the prokaryotic component whereas much less is known about the virome. However, recent viral metagenomics studies show that the intestinal virome in healthy mammals can be divided into three parts—the prokaryotic (bacteriophages), the archaeal, and the eukaryotic virome, see Fig 1 [19,20]. The eukaryotic virome comprises viruses infecting the host, endogenous viral elements, and viruses associated with other eukaryotic components of the ingesta.

Investigations of the porcine intestinal virome are so far limited [21–23]. Previous comparative analyses between healthy and diarrhoeic piglets are based on faecal samples and investigate piglets around weaning (19–30 days of age) [21,22] or at 6 weeks of age [23]. However, comparative analyses of the intestinal virome from healthy and diarrhoeic neonatal piglets have, to the best of the authors’ knowledge, not been made. Neither has the eukaryotic intestinal virome of healthy neonates been characterised in livestock.

The aim of the present study was to examine a potential involvement of previously described mammalian viruses in porcine neonatal diarrhoea of uncertain aetiology and to describe the intestinal eukaryotic virome in both healthy and diarrhoeic pigs. The current study presents comparative metagenomic data analysis of intestinal samples from 19 healthy and 50 diarrhoeic piglets from 10 herds in Sweden.

## Material and Methods

Samples were collected from animals from ten conventional herds (designated A to J, see Table 1) located in the middle of Sweden. All sampling procedures were approved by the Ethics Committee for Animal Experimentation, Uppsala, Sweden (permission number: C120/11).

**Table 1:** Descriptive data on selected herds (n = 10, A-J) and piglets (n = 69).

Herd	A	B	C	D	E	F	G	H	I	J
Type of production	Satellite	Farrow-to- finish	Piglet production	Piglet production	Satellite	Piglet production	Piglet production	Farrow-to- finish	Satellite	Farrow-to- finish
Sows in production	^a^	800	600	1100	^b^	160	500	120	^c^	250
Sanitation between batches	Washing + disinfection	Washing	Washing + disinfection	Washing	Washing + disinfection	Washing	Washing	Washing	Washing	Washing + disinfection
No of litters sampled	7	5	5	7	7	6	7	7	6	3
Age range of selected of piglets (days)^d^	1–2	1–6	1–3	<1–3	<1–2	<1–2	<1–3	<1–2	<1–3	<1–1
Sex of selected piglets	3♀, 4♂	3♀, 4♂	4♀, 3♂	7♀, 0♂	4♀, 3♂	5♀, 2♂	5♀, 2♂	5♀, 2♂	4♀, 2♂	5♀, 2♂
Time of visit	Jun+Sept 2011	Oct 2011	Nov 2011	Jan 2012	Feb 2012	Mar 2012	Mar 2012	Apr 2012	Apr 2012	May 2012

^a^ Farrowing on a weekly basis, 50 or 10 sows alternating every other week

^b^ Farrowing every two weeks, 44 sows per batch

^c^ Farrowing every seven or eight weeks, 72 sows per batch

^d^ Age was registered as <1 day (born the night before the visit), 1 day old (born the day before the visit), etc.

Recruitment of herds to the study was performed on a voluntary basis and all farmers gave their permission to conduct the study on their farms. This investigation is a part of a series of studies on NPD with uncertain aetiology. Pathological and bacteriological findings have been characterised previously [13,24] and are mentioned in the discussion.

### Sample collection

All herds were affected by NPD, despite routine vaccination of sows and gilts against ETEC. Two herds (A and E) were satellites in the same sow pool; two others (I and J) were located in proximity to each other and partially shared the same personnel. A total of 50 diarrhoeic animals and 19 healthy controls were included in the study; there were five diarrhoeic animals and two healthy controls from each herd (except for herd I where only one control piglet was obtained). Piglets originated from 60 different litters (three to seven per herd). Pigs treated with antibiotics were excluded. The 45 females and 24 males were all cross-breeds. Diarrhoeic pigs ranged in age from 0.5 to 6 days (median age, 1 day) and the controls from 1 to 5 (median age, 2 days), see Table 1.

The animals were transported alive to the Section of Pathology, Swedish University of Agricultural Sciences (SLU) where they were anaesthetised with an intramuscular injection of tiletamine and zolazepam, 6 mg/kg (Zoletil ^®^ 50, Virbac Animal Health, Carros Cedex, France) and euthanized by intracardiac injection of pentobarbital sodium, 100 mg/kg (Pentobarbital for Euthanasia 20% w/v solution for injection, Pharmasol Ltd, Andover, UK). Tissue specimens from the distal jejunum (40 cm proximal to the ileocaecal junction, including both intestinal tissue and contents) were collected immediately after euthanasia, snap frozen in liquid nitrogen and stored at -70°C.

### Sample preparation

Samples were prepared according to [24] with modifications. In brief, a 2.5 mm transverse section of the distal jejunum from each pig was placed in 1 ml PBS with 5% bovine serum albumin (BSA, Sigma Aldrich, St. Louis, MO, USA). The samples were subjected to three consecutive cycles of freeze-thawing on dry ice and homogenisation by the Omni TH Motor (Omni International, Kennesaw, GA, USA) using soft tissue Omni Tip^™^ Plastic Homogenizer Probes (Omni International). After homogenisation, samples were centrifuged for 10 min at 600 x *g*. The supernatants were collected and filtered through a 0.45 μm sterile filter (Merck Millipore, Billerica, MA, USA) and split into three fractions—one for extraction of viral RNA, one for extraction of viral DNA and one to be stored untreated at -70°C. Samples were kept on ice/dry ice slurry throughout the process. The RNA and DNA samples were treated with a nuclease cocktail containing TURBO^™^ DNase (2 U/μL; Thermo Fisher Scientific, Waltham, MA, USA), RNase Cocktail^™^ Enzyme Mix (RNase A; 500 U/mL) and RNase T1 (20,000 U/mL; Thermo Fisher Scientific) in 1x TURBO^™^ DNase Buffer. RNA and DNA were extracted as previously described [25]. Extracted RNA and DNA were stored at -70°C. A process control was prepared together with the clinical samples. This control contained PBS/5% BSA and was treated the same way as the samples, yielding one control each for RNA and DNA extractions. These controls were used to evaluate possible contaminants from the laboratory environment and reagents during preparation of the clinical specimens.

### Sequence Independent Single Primer Amplification

Sequence Independent Single Primer Amplification (SISPA) was performed according to Rosseel *et al*. 2013 [26]. Two mock metagenomes containing viruses from various families and genome types were used as positive controls—one for DNA viruses and one for RNA viruses. To check for contamination during the amplification, several non-template controls (NTCs) were included in every amplification set. The NTCs, process control, and positive controls, were processed next to the other samples as described below. In short, samples were tagged with two different SISPA tags (FR20RV-12N, 5’-GCCGGAGCTCTGCAGATATCNNNNNNNNNNNN-3’ and K-12N, 5’-GACCATCTAGCGACCTCCACNNNNNNNNNNNN-3’, respectively) in separate reactions. RNA samples were heated to 95°C for five minutes in the presence of the SISPA primer-tags and then put on ice. Samples were subsequently converted to cDNA using Superscript III (Thermo Fisher Scientific) according to manufacturer’s instructions. DNA samples were heated to 95°C for five minutes in the presence of the SISPA primer-tags and dNTPs, then put on ice. After 2 min, the Klenow Fragment (3’→ 5’ exo^−^) (New England Biolabs, Ipswich, MA, USA) was added and samples were incubated according to manufacturer’s instructions. SISPA was performed in two separate reactions for all samples, one with the FR20RV primer (5’-GCCGGAGCTCTGCAGATATC-3’) and one with the K primer (5’-GACCATCTAGCGACCTCCAC-3’) as previously described [26]. However, the polymerase was the KAPA HIFI HotStart ready mix (KAPA Biosystems, Wilmington, MA, USA).

Following PCR, the products were cleaned using the GeneJET PCR purification kit (Thermo Fisher Scientific) and subsequently subjected to cleavage using EcoRV (New England Biolabs). Cleaved products were repurified using the GeneJET PCR purification kit (Thermo Fisher Scientific) and quantified using the Qubit^™^ 2.0 Fluorometer (Thermo Fisher Scientific) and Qubit^®^ dsDNA HS Assay Kit (Thermo Fisher Scientific). Fraction size was checked by gel electrophoresis [27]. Finally, SISPA products from the DNA and RNA fractions with K and FR20RV primers were pooled per piglet and control sample (the positive control with mock metagenomes, the process control, and the NTCs from the SISPA reaction). Thereafter, samples from diarrhoeic piglets within the same herd were pooled, resulting in three samples per herd.

### Library preparation and sequencing

Sequencing was performed using the Ion Proton system (Thermo Fisher Scientific) at the National Genomics Infrastructure in Uppsala. 0.4 μg DNA from each sample was fragmented using the S2 system from Covaris (Covaris Inc., Woburn, USA). End repair and adaptor ligation were performed by the AB Library builder (Thermo Fisher Scientific). Samples were amplified according to the Ion Xpress^™^ Plus gDNA Fragment Library Preparation protocol and selected with a target size of 250–310 bp (Blue PippinTM, Sage Science, Beverly, MA, USA). Library size and concentration were assessed by a Bioanalyzer High Sensitivity Chip (Agilent Technologies, Santa Clara, CA, USA) and by the Fragment Analyzer system (Advanced Analytical, Ankeny, IA, USA). Samples were pooled together in sets of six, followed by template preparation using the Ion Proton^™^ Template OT2 200 v3 Kit on either the Ion OneTouch^™^ 2 system or the Ion Chef system (Thermo Fisher Scientific). Samples were then sequenced on the Ion Proton^™^ system using the Ion Proton^™^ Sequencing 200 v3 Kit on Ion PI v2 chips (200 bp read length, Thermo Fisher Scientific). The Ion Reporter system was running Torrent Suite 4.2.1 at the time of analysis.

### Bioinformatics

#### Quality control and filtering

Data in the form of de-multiplexed FASTQ files were acquired from the sequencing centre together with quality metrics for the sequencing runs. All FASTQ files were subjected to quality controls using PrinSeq version 0.20.4 [28]. For settings, a mean quality (Phred Quality score) [29] of 20 was used; sequences shorter than 110 bases were omitted. Following this, the datasets were mapped towards the host genome (Sus Scrofa domesticus genome, Genome assembly: Sscrofa10.2 (GCA_000003025.4) [30] using bowtie2 version 2.2.5 [31], to remove host sequences.

#### Taxonomic classification

**Fig 2:**
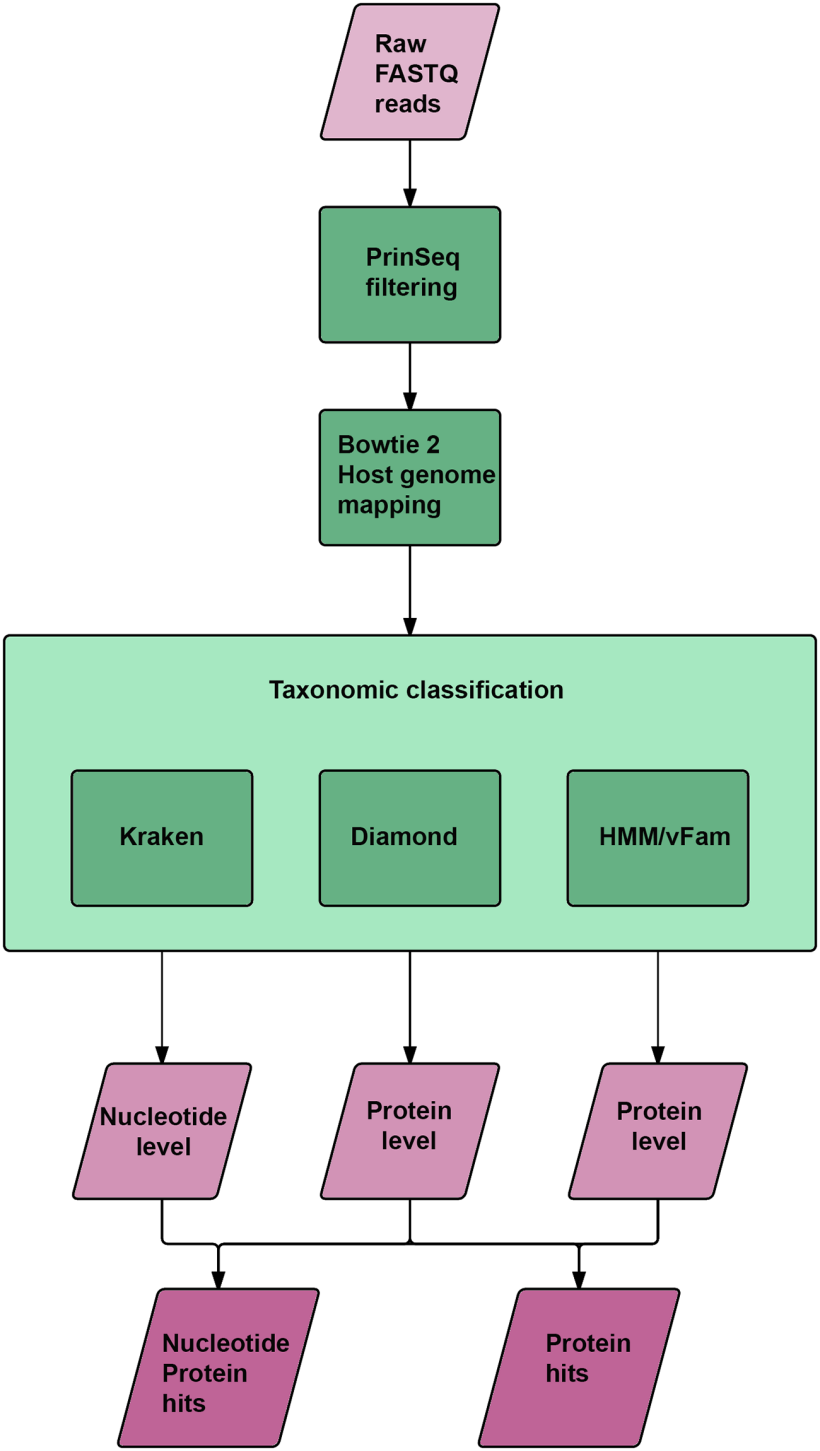
Schematic illustration of the bioinformatic pipeline. Raw data from the sequencing platform, in FASTQ format, was processed by PrinSeq to select high quality reads. The fractions of high quality reads were then mapped towards the porcine genome, using Bowtie 2, to remove host sequences. The unmapped reads were then classified by three different methods, Kraken, Diamond and HMM/vFam. The resulting three datasets were grouped in two datasets, one representing reads classified on nucleotide and protein level and one representing reads classified only on protein level.

Taxonomic classification was performed on read level using Kraken version 0.10.5-beta [32] and the BLASTx equivalent of Diamond (version 0.7.10) using the non-redundant (*nr*) database from NCBI (version of 2016-01-20) [33]. The same reads were used for predicting protein sequences with FragGeneScan (FGS) 1.19, and the resulting sequences were scanned, using HMMER3, for the viral profiles Hidden Markov Models (HMM) of the vFam database (February 2014 release) [34,35]. The Kraken database used for the classification is an expanded in-house database covering all sequences from the viral (VRL) and phage (PHG) divisions of GenBank (NCBI), as well as the bacterial and archaeal sequences from RefSeq [36]. See Fig 2 for an overview of the data analysis pipeline. The results are only presented descriptively since the individual samples from the healthy animals were not directly comparable with the pooled diarrhoeic samples.

## Results

Sequencing data averaged 220 Mbases of data for the clinical samples with a mean read length of 185 bases; see Table 2 for the metrics of individual samples. The positive control generated 6.3 Gbases of high quality (52% good quality sequences) with a mean read length of 194 bases after filtering. In contrast, the NTC generated only 124 Mbases (21.74% good quality sequences) with a mean read length of 194 bases. In the positive control, all viruses included in the mock metagenome were detected whereas the NTC rendered no relevant hits. The process control contained 204 Mbases of good quality (26% good quality sequences) with a mean length of 180 bases; no mammalian viruses were detected. All clinical samples contained endogenous viral elements. Sequences classified as PERV were absent in the NTC and the process control. Data for the clinical samples are available at the European Nucleotide Archive, study accession number PRJEB11519.

**Table 2:** Overview of sequencing results and initial filtering and read classification.

Sample ID	No. reads passing quality check^a^	Average read length (bases)	Reads mapping towards host genome^b^	Sequences classified as bacterial with Kraken^c^	Unclassified sequences with Kraken	Sequences classified as bacterial with Diamond^d^	Unclassified sequences with Diamond
1A	606,302	183.93	44.06%	4.75%	26.26%	10.48%	26.31%
2A	2,504,629	185.42	76.00%	2.61%	60.39%	14.33%	21.11%
Pool A	369,295	180.11	96.84%	30.31%	43.58%	50.16%	29.43%
1B	1,539,095	185.02	60.28%	2.39%	85.17%	32.44%	25.02%
2B	2,136,923	183.3	64.20%	2.88%	84.01%	34.49%	28.80%
Pool B	516,471	181.6	91.04%	9.02%	47.34%	15.83%	47.13%
1C	1,682,302	182.23	60.28%	0.54%	60.17%	7.27%	4.55%
2C	594,205	183.34	64.20%	2.43%	73.94%	12.87%	7.76%
Pool C	501,732	175.93	94.85%	64.17%	29.12%	67.57%	21.50%
1D	1,911,914	179.65	79.81%	10.45%	78.21%	74.17%	20.84%
2D	1,038,254	179.78	92.82%	46.58%	42.90%	57.47%	18.17%
Pool D	457,987	176.29	98.43%	19.42%	74.60%	63.43%	23.19%
1E	1,818,102	191.14	91.77%	3.35%	77.33%	23.58%	16.97%
2E	1,108,215	192.44	99.26%	8.75%	79.66%	57.66%	25.37%
Pool E	769,456	188.86	97.69%	52.05%	46.17%	81.98%	14.40%
1F	1,351,927	190.39	94.95%	2.80%	96.19%	68.44%	23.11%
2F	843,996	188.13	94.82%	4.38%	89.22%	44.80%	32.04%
Pool F	1,076,708	190.21	94.94%	85.17%	12.59%	57.85%	40.29%
1G	1,737,954	185.42	89.03%	2.07%	74.97%	20.60%	12.28%
2G	1,563,659	184.27	72.58%	2.25%	54.16%	13.88%	30.46%
Pool G	930,505	189.72	98.60%	37.93%	60.67%	76.86%	22.22%
1H	1,318,250	186.03	87.97%	1.72%	97.89%	51.89%	44.26%
2H	1,855,750	186.62	91.99%	2.18%	97.20%	48.44%	45.13%
Pool H	452,993	187.39	96.90%	12.97%	79.44%	57.70%	26.73%
1I	872,958	187.4	95.01%	2.97%	95.88%	62.67%	27.38%
Pool I	926,498	184.34	98.94%	31.56%	58.73%	65.23%	22.82%
1J	1,763,112	186.24	69.27%	3.82%	45.46%	15.32%	26.86%
2J	1,756,153	184.99	51.75%	3.43%	59.45%	17.16%	18.06%
Pool J	674,860	183.99	96.17%	35.55%	25.29%	35.07%	43.97%
NTC	638,932	194	18.19%	21.60%	76.87%	21.60%	76.87%
Process control	1,132,200	180	21.93%	12.58%	85.42%	61.81%	38.09%

^a^ Reads passing the PrinSeq filtering

^b^ Percentage of good quality reads mapping towards porcine host genome using Bowtie 2.

^c^ Percentage of good quality reads classified towards bacterial RefSeq sequences included in the Kraken database.

^d^ Percentage of good quality reads classified towards bacterial protein sequences in the NCBI non-redundant (nr) database.

### Detection of mammalian viruses in porcine distal jejunum

Table 3 reports classification of viral sequences on nucleotide level (Kraken) and protein level (Diamond and HMM/vFam). Viral families detected on both nucleotide and protein level included sequences belonging to eight different virus families infecting mammals: *Adenoviridae*, *Anelloviridae*, *Astroviridae*, *Caliciviridae*, *Circoviridae*, *Parvoviridae*, *Picornaviridae*, and *Reoviridae*. Viral sequences in healthy samples included hits towards *Adenoviridae* (n = 3), *Anelloviridae* (n = 1), *Astroviridae* (n = 1), *Caliciviridae* (n = 1), *Circoviridae* (n = 8), *Parvoviridae* (n = 2), *Picornaviridae* (n = 10), and *Reoviridae* (n = 2). Viral sequences detected in pooled diarrhoeic samples were *Anelloviridae* (n = 1), *Circoviridae* (n = 1), *Picornaviridae* (n = 4), and *Reoviridae* (n = 2). More than one mammalian virus family was present in ten out of 19 samples from healthy piglets and in two out of 10 pooled samples from diarrhoeic piglets. In four samples from individual healthy piglets (2A, 1B, 2B, 1C, and 1G), sequences belonging to three different mammalian virus families were detected. See Table 4 for a summary of the virus families found in healthy and diarrhoeic samples respectively.

**Table 3:** Overview of samples and viruses detected at family level in the distal jejunum of neonatal piglets with and without diarrhoea.

Sample ID	Herd	Health status	Age (Days)^a^	Mammalian viruses detected (No. of reads)^b^	Mammalian viruses detected (No. reads)^c^	Mammalian viruses detected (No. reads)^d^
				Kraken	Diamond	*HMM/*vFam
1A	A	Healthy	2	*Picornaviridae* (57891)	*Picornaviridae* (27997)	*Picornaviridae (25840)*
				N/A	N/A	*Parvoviridae (8)*
2A	A	Healthy	1	*Picornaviridae* (174352)	*Picornaviridae* (106330)	*Picornaviridae (95786)*
				*Caliciviridae* (184)	N/A	*Caliciviridae (4480)*
				*Parvoviridae* (13)	N/A	*Parvoviridae (2)*
				N/A	*Circoviridae* (4)	*Circoviridae (27)*
Pool A	A	Diarrhoeic	1	*Reoviridae* (2)	N/A	*Reoviridae (1)*
1B	B	Healthy	4	*Picornaviridae* (1201)	N/A	*Picornaviridae (750)*
				*Reoviridae* (28874)	*Reoviridae* (21161)	*Reoviridae (18127)*
				*Circoviridae* (88)	*Circoviridae* (85)	*Circoviridae (213)*
				N/A	N/A	*Adenoviridae (18)*
2B	B	Healthy	5	*Picornaviridae* (52101)	*Picornaviridae* (27870)	*Picornaviridae (29473)*
				*Reoviridae* (6)	*Reoviridae* (6)	*Reoviridae (52)*
				*Circoviridae* (946)	*Circoviridae* (731)	*Circoviridae (389)*
				N/A	N/A	*Adenoviridae (33)*
Pool B	B	Diarrhoeic	3.2	*Picornaviridae* (880)	*Picornaviridae* (460)	*Picornaviridae (605)*
				N/A	N/A	*Caliciviridae (48)*
				*Reoviridae* (690)	*Reoviridae* (1184)	*Reoviridae (1372)*
1C	C	Healthy	3	*Picornaviridae* (1615)	*Picornaviridae* (798)	*Picornaviridae (807)*
				*Astroviridae* (23)	*Astroviridae* (15)	*Astroviridae (27)*
				*Circoviridae* (10)	*Circoviridae* (6)	*Circoviridae (53)*
				N/A	N/A	*Caliciviridae (42)*
2C	C	Healthy	1	N/A	*Circoviridae* (2)	*Circoviridae (45)*
Pool C	C	Diarrhoeic	1.9	N/A	N/A	*N/A*
1D	D	Healthy	1	*Circoviridae* (1)	*Circoviridae* (3)	*Circoviridae (998)*
				*Adenoviridae* (1)	*Adenoviridae* (1)	*Adenoviridae (10)*
2D	D	Healthy	1	N/A	*Circoviridae* (19)	*Circoviridae (47)*
Pool D	D	Diarrhoeic	1.2	N/A	N/A	*N/A*
1E	E	Healthy	1	*Anelloviridae* (321)	*Anelloviridae* (151)	*Anelloviridae (82)*
2E	E	Healthy	2	N/A	N/A	*Circoviridae (16)*
Pool E	E	Diarrhoeic	1.1	*Anelloviridae* (12)	*Anelloviridae* (10)	*Anelloviridae (3)*
				*Circoviridae* (1)	*Circoviridae* (1)	*Circoviridae (4)*
1F	F	Healthy	3	*Picornaviridae* (6)	*Picornaviridae* (1)	*Picornaviridae (9)*
				*Circoviridae* (51)	*Circoviridae* (36)	*Circoviridae (64)*
				N/A	N/A	*Caliciviridae (5)*
2F	F	Healthy	2	*Picornaviridae* (1)	N/A	*Picornaviridae (6)*
				N/A	N/A	*Circoviridae (21)*
Pool F	F	Diarrhoeic	2.1	*Picornaviridae* (674)	*Picornaviridae* (411)	*Picornaviridae (365)*
				N/A	N/A	*Caliciviridae (108)*
				N/A	*Picobirnaviridae* (4)	*N/A*
1G	G	Healthy	2	*Parvoviridae* (222)	*Parvoviridae* (186)	*Parvoviridae (10)*
				*Picornaviridae* (1755)	*Picornaviridae* (955)	*Picornaviridae (936)*
				N/A	N/A	*Caliciviridae (97)*
				*Circoviridae* (52)	N/A	*Circoviridae (39)*
2G	G	Healthy	2	*Picornaviridae* (183389)	*Picornaviridae* (85080)	*Picornaviridae (92805)*
				*Circoviridae* (9)	*Circoviridae* (24)	*Circoviridae (121)*
				N/A	N/A	*Caliciviridae (16108)*
Pool G		Diarrhoeic	2.6	*Picornaviridae* (2)	*Picornaviridae* (2)	*Picornaviridae (2)*
1H	H	Healthy	2	N/A	*Circoviridae* (1)	*Circoviridae (11)*
2H	H	Healthy	3	*Picornaviridae* (20)	*Picornaviridae* (15)	*Picornaviridae (20)*
				N/A	N/A	*Circoviridae (323)*
Pool H	H	Diarrhoeic	2.6	N/A	N/A	*Reoviridae (5)*
1I	I	Healthy	3	*Adenoviridae* (1)	*Adenoviridae* (1)	*Adenoviridae (29)*
				*Circoviridae* (6)	*Circoviridae* (12)	*Circoviridae (57)*
Pool I	I	Diarrhoeic	1.9	*Picornaviridae* (133)	*Picornaviridae* (69)	*Picornaviridae (64)*
				N/A	N/A	*Caliciviridae (2)*
				N/A	*Circoviridae* (1)	*N/A*
1J	J	Healthy	1	N/A	*Circoviridae* (6)	*Circoviridae (25)*
2J	J	Healthy	1	N/A	*Circoviridae* (25)	*Circoviridae (24)*
				N/A	N/A	*Parvoviridae (3)*
				*Adenoviridae* (1)	N/A	*Adenoviridae (2)*
Pool J		Diarrhoeic	0.8	N/A	N/A	*Circoviridae (4)*

^a^ Age in days for individual piglets. For pooled samples, the average age is given.

^b^ Reads classified as homologous to viral nucleotide sequences by Kraken reported on family level.

^c^ Reads classified as homologous to viral proteins using Diamonds BLASTx settings reported on family level.

^d^ Reads classified as homologous to viral protein families in the vFam database reported on viral family level.

**Table 4:** Number of samples from healthy piglets (n = 19) and pooled diarrhoeic samples (n = 10) positive for virus families known to infect mammals.

Virus families	Healthy samples		Diarrhoeic pools	
	n	%	n	%
*Adenoviridae*	3	16	0	0
*Anelloviridae*	1	5	1	10
*Astroviridae*	1	5	0	0
*Caliciviridae*	1	5	0	0
*Circoviridae*	8	42	1	10
*Parvoviridae*	2	11	0	0
*Picornaviridae*	10	53	4	40
*Reoviridae*	2	11	2	20

^a^Detected mammalian viruses at family level. Viruses detected only on protein level is not included.

In several samples classification of reads to viral families occurred only on protein level. In five samples both Diamond and HMM/vFam detected *Circoviridae*. In addition, Diamond detected *Circoviridae (*n = 1), *Picobirnaviridae* (n = 1) and HMM/vFam detected *Adenoviridae* (n = 2), *Circoviridae* (n = 4), *Caliciviridae* (n = 7), *Parvoviridae* (n = 2), and *Reoviridae* (n = 1).

The most common virus family detected was *Picornaviridae*, which was present in 14 out of 29 samples followed by *Circoviridae* reported in 9 out of 29 samples. The majority of the *Picornaviridae* sequences were classified on nucleotide level as species *Aichivirus C*. Sequences classified as *Circoviridae* in samples (n = 9) were interestingly absent in all diarrhoeic samples but one (E) and were identified on a nucleotide level as po-circo-like virus 21, 22, 51, porcine stool-associated circular virus 5, and fur seal faeces associated circular DNA virus. In addition, some unassigned small circular ssDNA viruses were found in seven healthy piglets. *Reoviridae* sequences included hits on nucleotide level against the species *Rotavirus A* and *Rotavirus C*. Both *Rotavirus A* and *C* were detected in two samples (1B and pool B) and only *Rotavirus A* in one sample (Pool A). In three samples (1B, 2B, and 2E) 18, 20 and 5 reads, respectively, were classified on nucleotide level as the dsDNA virus immunodeficiency associated stool virus (IAS virus).

## Discussion

To the best of our knowledge, this is the first comparative metagenomics study of the eukaryotic virome in the intestine of healthy and diarrhoeic neonatal piglets. Furthermore, it is the first study to describe the eukaryotic intestinal virome in healthy neonatal livestock. It was not possible to associate a particular virus family with NNPDS. Among the mammalian viruses found in diarrhoeic piglets—*Anelloviridae*, *Circoviridae*, *Picornaviridae* and *Reoviridae—*only *Reoviridae* includes well established viral enteropathogens causing piglet diarrhoea [2,37,38]. *Rotavirus A* and *C* were found in herd B (both in samples from healthy piglets and in the pooled diarrhoeic sample) and *Rotavirus A* in the pooled diarrhoeic sample from herd A. Previous investigations of the same animals have shown that common bacteriological enteropathogens were either absent (*Clostridium perfringens* type C), rare (enterotoxigenic *Escherichia coli*—only detected in two animals from herd A and E, respectively) or unrelated to the presence of diarrhoea (*Clostridium perfringens* type A and *Clostridium difficile*) [13]. However, 60% of the diarrhoeic animals in herd B, C, F, H, I, and J were found to be colonised in the small intestine by enteroadherent *Enterococcus (E*.*) hirae* [39]. Small intestinal villus atrophy was observed in five diarrhoeic piglets in total and small intestinal epithelial lesions 13. The majority of these animals were colonised by *E*. *hirae* (4 of 5 with villous atrophy and 10/13 with epithelial lesions). Although it is not possible to relate viral findings to individual piglets, two of the piglets with small intestinal atrophy originated from herd B (none from herd A) and it is hence possible that rotavirus contributed to the intestinal pathology seen in these animals. However, overall the results of this study do not show a major contribution of known viruses to the investigated diarrhoea.

### The composition of mammalian viruses in the intestine of healthy and diarrhoeic neonatal piglets

A number of different virus families known to infect mammals were detected in the present study (*Adenoviridae*, *Anelloviridae*, *Astroviridae*, *Caliciviridae*, *Circoviridae*, *Parvoviridae*, *Picornaviridae*, and *Reoviridae*). These findings agree with recent studies on the porcine faecal virome indicating that the general composition of mammalian viruses in the porcine intestine is similar, despite differences in geographical location and age of the sampled pigs [21–23,40–42]. A notable difference, however, is the absence of the coronaviruses TGEV and PEDV in our study. This dissimilarity was expected since TGEV and PEDV have never been detected in Sweden.

Comparisons of the faecal virome between healthy and diarrhoeic pigs in a larger number of animals have so far been limited to two studies investigating piglets at an age of 19–30 days [21,22]. Similar to the present study, putative co-infections were common. Zhang *et al*. (2014) [21] reported a mean of 2 viral families in the faeces of healthy pigs (n = 29) compared to 3.8 viral families in diarrhoeic pigs (n = 27). Similarly, Shan *et al*. (2011) [22] found an average of 2.75 viral families in faecal samples from healthy pigs (n = 24) and 3.7 in diarrhoeic pigs (n = 12). When comparing our results with these previous studies, it should be noted that we used intestinal tissue instead of faeces. In addition, the diarrhoeic samples were pooled in the present study (each pool containing samples from five pigs) and thus the number of viral families in individual diarrhoeic piglets is unknown. Interestingly, compared to previous studies, we found an overall smaller number of different virus families both in samples from healthy individual piglets (0–3, mean = 1.5) and in pooled samples from diarrhoeic piglets (0–2, mean = 0.8). This difference is likely due to the lower age of the piglets in the present study (median age 1 day for diarrhoeic piglets and median age 2 days for controls). An additional factor that could have contributed to the low number of viruses in neonatal piglets is the passive immunity received through colostrum.

Another contradiction with previous studies is that we found a greater number of different virus families in healthy piglets compared to the pooled diarrhoeic samples. Two families or more were detected in 2/10 pooled diarrhoeic samples compared to 10/19 from healthy piglets. This could be related to age, as healthy piglets were slightly older. Alternatively, the diarrhoea could have led to a decreased complexity of the intestinal virome. These results call for further studies including analysis of individual diarrhoeic pigs.

### Detection of *Aichivirus C*, eukaryotic small circular DNA viruses, and IAS virus

The most common findings were sequences belonging to *Picornaviridae* and eukaryotic circular single-stranded DNA viruses classified as *Circoviridae*. The majority of *Picornaviridae-s*equences—detected in 10/19 healthy piglets and in 4/10 pooled diarrhoeic samples—were classified at species level as *Aichivirus C*. A number of studies have reported a high prevalence of *Aichivirus C* in faeces from both healthy and diarrhoeic piglets [43–45] and an association with diarrhoea is hence controversial. The transmission route of *Aichivirus C* is thought to be faecal-oral, but the virus has also been detected in sera only three days after birth, demonstrating its capacity to escape the gastrointestinal tract [46]. This, together with the present detection of *Aichivirus C* in the intestine within 24–48 h from birth, calls for further investigations of possible transplacental or lactogenic transmission routes.

Several sequences in the present study were identified as unclassified *Circoviridae* (po-circo-like virus 21, 22, 51 and fur seal faeces associated circular DNA virus) or as unassigned small circular ssDNA viruses. These newly discovered unassigned small circular ssDNA viruses, are highly divergent viruses encoding a well-conserved replication initiator protein (Rep) involved in rolling circle replication [47]. Recently, circular Rep -encoding ssDNA (CRESS-DNA) viruses have been identified in a variety of samples including insects, plants, sewage and mammalian faeces, but the origin and host of these viruses are presently unclear [47–50]. Interestingly, we found sequences classified as CRESS-DNA viruses in only clinically healthy piglets. These results agree with the hypothesis that these viruses might be of dietary origin or possibly a commensal part of the eukaryotic virome [49,51].

Interestingly, a small number of sequences from three healthy piglets shared similarities with immunodeficiency -associated stool virus (IASV). This large dsDNA virus has previously been reported in only one study on diarrhoea in humans with advanced-stage HIV infection [52]. The IAS virus is highly divergent from previously characterised viruses and further characterisation of our sequences is required to establish if this in fact is an eukaryotic virus.

### Considerations for the use of viral metagenomics in the analysis of clinical samples

As this study relied on a viral metagenomics approach for investigating the virome, the quality of the sequence reads is highly important. The present study generated an average of 220 Mbases of good-quality data per clinical sample, with a mean read length of 185 bases. The NTC and process control followed the same pattern as the clinical samples, but at an analysis level, reported no significant hits towards known viral families. However, the positive control generated 6.3 Gbases of data.

Endogenous viral elements (EVE) were detected within all samples. However, no porcine EVE was detected within the process control and the NTC. In the positive control, porcine EVE sequences were detected which is probably due to the use of porcine cell lines for culturing viruses.

All samples were subjected to three analytical methods suited for detecting sequences in metagenomics datasets, Kraken, Diamond and HMM. As Kraken is only detecting similar sequences at a nucleotide level, two additional methods were used for detecting more distant homologues; the BLASTx equivalent of Diamond and HMMER3 for scanning predicted protein sequences (using FGS) for viral profiles (vFam). Together all three methods enable not only detection of viruses on species level but also a broader detection on family level. As a consequence, some additional viral families were detected only by protein homology. Diamond and HMM/vFam detected putative *Circoviridae* sequences in five different samples. By classification with Diamond, *Picobirnaviridae* homologues were classified on species level to porcine picobirnavirus, however this concerns only four reads in one diarrhoeic sample. Porcine picobirnavirus has frequently been detected in pigs but its association to diarrhoea is uncertain [53,54]. Moreover, classifications with only HMM/vFam included additional hits towards *Caliciviridae*, *Adenoviridae*, *Reoviridae*, *Parvoviridae* and *Circoviridae*, indicating the presence of proteins homologous to proteins from viruses belonging to these families. However, the results did not show any consistent pattern in relation to the piglets’ health status.

### The mammalian intestinal virome in neonates

The present study showed that viral infections were present as early as 24–48 h after birth. The presence of several different eukaryotic viruses in the intestine of healthy piglets suggests that some of the viruses may be regular constituents of the porcine intestinal virome, whereas others may be capable of causing disease under certain circumstances. As an increasing number of eukaryotic viruses are being detected in mammalian tissues without any overt signs of disease in the host, the concept of commensal virus infections has been proposed [19,20]. It has recently also been suggested that eukaryotic viruses may be important for gut homeostasis as the presence of a common enteric RNA virus was able to replace the beneficial functions of commensal bacteria in mice [55].

Studies on the neonatal intestinal virome in mammals younger than a week of age are limited to one recently published study in humans [20]. The number of eukaryotic viruses was low in the first week of life (similar to our study) and thereafter the complexity increases over time. This is in agreement with the development of the porcine intestinal virome, based on the present and previous studies [21,22].

## Conclusions

Given previous knowledge of gastrointestinal viruses and their load in faeces as well as the intestinal mucosa during clinical disease, it is highly unlikely that a known virus would have remained undetected [56,57]. Our data did not support the hypothesis that NNPDS is caused by known mammalian viruses. However, the metagenomics analyses resulted in a preliminary list of viruses present in diarrhoeic and healthy neonatal piglets in Sweden, providing a starting point for future investigations. The study also concludes that viruses are present in the intestine as early on as 24–48 h after birth, indicating immediate infection post-partum, alternatively, transplacental infection. Nevertheless, the findings do require *in vitro* validation as well as full genomic characterisation to fully establish the diversity of the porcine neonatal virome. Further, the smaller number of different viruses found in this study on neonatal pigs compared to previous studies on pigs around weaning [21,22] support recent findings in humans demonstrating a progressive establishment of the intestinal virome [20]. These findings warrant further longitudinal studies on the early-life dynamics of the developing intestinal virome.
